# Using triple radio-immunotherapy to overcome cancer immunotherapy resistance

**DOI:** 10.20892/j.issn.2095-3941.2023.0268

**Published:** 2023-11-16

**Authors:** Zengfu Zhang, Xiaodong Zhang, Dawei Chen

**Affiliations:** 1 Department of Radiation Oncology, Shandong University Cancer Center, Jinan 250117, China; 2 Department of Radiation Oncology, Shandong Cancer Hospital and Institute, Shandong First Medical University and Shandong Academy of Medical Sciences, Jinan 250117, China

Cancer is a widespread public health issue and has become a leading cause of death in most countries. In recent decades, immunotherapy, particularly the use of immune checkpoint inhibitors (ICIs), has altered the clinical practice of cancer treatment and evolved into a first-line clinical strategy. ICIs significantly improve clinical outcomes and provide long-term benefits. However, several barriers limit the efficacy of cancer immunotherapy. First, ICIs as a monotherapy have limited effects, and only a minority of patients experience durable survival benefits. Second, a proportion of patients who initially show favorable responses gradually develop acquired resistance. Finally, current ICIs are directed primarily to therapy-naïve patients, and alterations in the immune microenvironment after treatment are not considered. Therefore, existing ICIs might be not the most suitable therapy for patients who have received prior treatments. In addition, these ICIs might not satisfy the requirement for personalized therapies. To address these barriers, researchers have extensively investigated combination treatment strategies. Preclinical and clinical data have shown that radiotherapy plus immunotherapy is an effective combination strategy to improve systemic anti-tumor response by amplifying the immunomodulatory effects. Many attempts have been made to combine radiotherapy with immunotherapy. Herrera and colleagues^1^ have found that a model named “RACIM” comprising low-dose radiotherapy plus immunotherapy has synergistic effects on tumor killing in patients with poor immune infiltration. Our team have conducted a more in-depth study of radiotherapy combined with ICIs (iRT), given that novel radiotherapy modalities, such as low dose irradiation (LDIR) and alternative immune targets, may provide a triple radio-immunotherapy that more powerfully increases treatment efficacy.

## Current status and challenges of iRT

The proposal of iRT was based on deeper understanding of the rationale of radiotherapy-induced immune effect. The opinion that radiotherapy is merely a simple local therapy for direct DNA damage to tumors has already changed. Currently, radiotherapy is generally accepted to exert a systemic anti-tumor response relying on immunostimulatory effects. One representative example is the abscopal effect. Although, in some cases, the abscopal effect cannot be observed and evaluated, molecular and genetic alterations have been found to appear before tumor regression. Hence, a comprehensive understanding of the abscopal effect, from the macroscopic, molecular, and genetic levels, has been suggested to be necessary^2^. In the PEMBRO-RT trial^3^, the tumor microenvironment (TME) after iRT has been found to exhibit an immunological abscopal effect, with increased infiltration of cytotoxic T cells and immune molecules. The mechanisms underlying anti-tumor immunity triggered by radiotherapy may be attributable to the *in situ* vaccination effect. Radiotherapy elicits immunogenic cell death of tumor cells, thus resulting in the release of numerous tumor-associated antigens and damage-associated molecular patterns (DAMPs). These antigens are captured and presented by dendritic cells and macrophages, thus further activating the adaptive anti-tumor immune response. The released DAMPs can lead to an adaptive anti-tumor response and augment the innate anti-tumor immune response. Evidence has demonstrated the substantial potential of radiotherapy to improve the efficacy of ICIs.

However, to maximize synergistic effects, many unresolved issues must be addressed in the selection of an optimal radiotherapy regimen, including the dose, fraction, and optimal time to intervene.

Many studies have also discussed the optimal radiotherapy dose for immune activation. Generally, the immune activation effect increases with increasing radiotherapy dose. Vanpouille-Box and colleagues^4^ have found that, in different cancer cells, radiation doses above 12–18 Gy decrease immunogenicity by inducing the DNA exonuclease Trex1 and degrading DNA accumulated in the cytoplasm. Interestingly, high-dose radiotherapy above 15 Gy has been found to cause tumor cell apoptosis and senescence, on the one hand, and to activate immune suppressive cells that inhibit the anti-tumor immune response, on the other hand. Furthermore, preclinical studies in animal models of breast cancer and colorectal cancer have shown that fractionated 3 × 8 Gy is more likely to promote the occurrence of the abscopal effect than a single 20 Gy dose^5^. Another study by Mittal^6^ has indicated that, in a mutant HKP1 (KrasG12D p53^−/−^) mouse lung cancer model, a 4 Gy × 3 radiotherapy regimen, compared with low-dose radiotherapy (LDRT) and hypofractionated radiotherapy (HFRT), achieves better tumor suppression and survival improvement in combination with PD-1 antibody treatment. Therefore, a single high dose may not be the best option for activating the body’s immunity.

The timing of appropriate radiation therapy in combination therapy is also crucial. Here, we discuss NCT05348668, a phase II multi-center study, in detail, as an example. That study selected patients with oligometastatic driver gene-negative non-small cell lung cancer (NSCLC), and explored whether a combination of radiotherapy and immunotherapy after immunotherapy involving mixed-dose fractionated radiotherapy might bring clinical benefits to patients.

Previous research conducted by the authors’ team indicated that approximately 50% of patients cannot benefit from pembrolizumab plus radiotherapy, although the best abscopal response rate of combination cohort is significantly higher than that of pembrolizumab alone^7^. Although several preclinical research and clinical trials have sought to address these obstacles, no consensus has been reached regarding selection of an optimal radiotherapy modality. Theoretically, HFRT, particularly stereotactic body radiation therapy (SBRT), may trigger massive release of tumor antigens and reprogram the TME, thereby enhancing anti-tumor immunity, whereas the effect of anti-tumor immunity is limited when conventional fractionated radiotherapy (CFRT) is used. The authors demonstrated better responses and outcomes of HFRT combined with pembrolizumab in patients with metastatic NSCLC through a pooled analysis of 2 clinical trials^7^. Furthermore, our team’s another study has indicated that SBRT combined with immunotherapy, compared with CFRT, yields improvements in response rates and progression-free survival^8^. A further analysis revealed that the underlying mechanism can be explained by the protective function of SBRT toward lymphocytes, thus potentially affecting abscopal responses and outcomes^9^. In addition, CFRT has been found to significantly promote local relapse and lung metastasis by altering the status of tumor-associated macrophages and tumor-associated neutrophils. However, given tumor heterogeneity and the immunosuppressive TME trained by tumor cells, multisite radiotherapy may achieve better systemic disease control in patients with metastatic cancer. Preliminary clinical data have verified the safety and efficacy of multisite SBRT followed by pembrolizumab^10^, or with concurrent nivolumab and ipilimumab^11^, in patients with advanced solid tumors. Although the results have been encouraging, more real world data are needed to confirm the findings, because of the unavoidable additive toxicity. Thus, novel intervention strategies should be investigated to improve efficacy while exploring the optimal combination modality for iRT; hence, triple radio-immunotherapy was proposed by the authors.

## Using LDIR and alternative immune targets to boost the efficacy of iRT

Given the limited efficacy and resistance of iRT, adding a new therapy to constitute triple radio-immunotherapy should be considered, according to aspects of radiotherapy and immunotherapy. Two appropriate partners for iRT are LDIR and alternative immune targets.

First, in the era of multisite radiation, LDIR may be a favorable choice for clinical practice, because of its advantages of safety and its unique role of turning “hot” tumors. In this triple therapy modality, the combination of high and low dose radiotherapy has unique immunomodulatory effects that achieve maximal enhancement of the efficacy of ICIs. The authors have used a combination of high and low dose gradient radiotherapy. Similarly, Welsh^12^ has also reported this strategy, called the “RadScopal” technique. Several studies using unconventional doses have suggested that LDIR can reprogram an immunosuppressive TME or a TME with minimal lymphocyte infiltration, thereby sensitizing ICIs^1,12^. The underlying mechanisms may include increasing infiltration of CD4^+^ effector T cells, promoting M1 macrophage polarization, enhancing nature killer cell infiltration and cytotoxic function, and decreasing levels of immunosuppressive cytokines. Moreover, LDIR can promote normalization of tumor vessels and lead to an inflammatory response providing a more favorable microenvironment. Thus, on the basis of the effects of *in situ* vaccination induced by HFRT, LDIR eliminates obstacles including vessel barriers and an immunosuppressive TME, thereby enabling effector immune cells to infiltrate and enhance the tumoricidal effect. Notably, the triple radio-immunotherapy comprising gradient radiotherapy plus ICIs has been preliminarily tested in preclinical animal models and clinical trials. Yin and colleagues^13^ have demonstrated that LDIR of metastatic sites increases T cell-attracting chemokines and enhances infiltration of CD8^+^ effector T cells, thus improving the abscopal effect induced by HFRT plus immunotherapy. Similarly, early findings from a phase II trial have indicated that LDIR with high-dose radiotherapy may achieve better lesion-specific control than high-dose radiotherapy alone for patients refractory to immunotherapy. These data have demonstrated that triple radio-immunotherapy may augment the systemic anti-tumor response, and that gradient radiotherapy may have profound effects on overcoming anti-PD-1 resistance and improving efficacy. **Table 1** summarizes ongoing clinical trials using gradient radiotherapy and ICIs and shows the relevant conditions, radiotherapy modalities, and immune targets.

**Table 1:** Current clinical trials on gradient radiotherapy combined with ICIs, according to ClinicalTrials.gov.

NCT number	Conditions	Gradient RT	ICIs
NCT03085719	Head and neck cancer	HFRT+LDIR	Pembrolizumab (PD-1)
NCT05755009	Metastatic cancer	HFRT+LDIR	Envafolimab (PD-L1)
NCT05547282	Cancer	CFRT+LDIR	Nivolumab (PD-1) Pembrolizumab (PD-1) Troripalimab (PD-1) Camrelizumab (PD-1)
NCT05650216	Esophageal squamous cell carcinoma	CFRT+LDIR	Camrelizumab (PD-1)
NCT03812549	Stage IV NSCLC	SBRT+LDIR	Sintilimab (PD-1)
NCT04917770	Nasopharyngeal carcinoma	SBRT+LDIR	Sintilimab (PD-1)
NCT05088889	Stage IV pancreatic cancer	SBRT+LDIR	Nivolumab (PD-1) Ipilimumab (CTLA-4)
NCT02444741	Stage IV NSCLC	SBRT+LDIR	Pembrolizumab (PD-1)
NCT05733156	Neoplasms, secondary malignant neoplasms	SBRT+LDIR	Not mentioned
NCT05978193	Esophageal squamous cell carcinoma, esophageal cancer, metastatic esophageal squamous cell carcinoma	CFRT+LDRT	PD-1 inhibitor
NCT05906329	Cancer	CFRT+LDRT	Not mentioned
NCT05348668	Stage IV NSCLC	LDIR+HFRT	Pembrolizumab (PD-1) Tislelizumab (PD-1)

Second, the therapy-altered TME and the emergence of novel immune targets may provide more opportunities for achieving anti-tumor effects. A series of studies have demonstrated that, regardless of therapy type, the TME may greatly change. For instance, radiotherapy shapes an inflammatory microenvironment to remodel the immune context through DNA damage and activation of the cyclic GMP-AMP synthase-stimulator of interferon genes (cGAS-STING) pathway. Anlotinib, an anti-angiogenic drug, has been found to enhance the cytotoxicity and proliferation ability of CD8^+^ T cells, and sensitize cells to radiotherapy by activating the cGAS/STING pathway^14^. Therefore, using DNA damage repair inhibitors and a cGAS-STING agonist to target the 2 key biological processes may augment the efficacy of iRT. Another approach to improve the efficacy of iRT is directed at other immune cell checkpoints. A recent study has reported that radiotherapy in combination with CD47 and PD-1 blockade enhances the macrophage-mediated abscopal effect through inhibiting the classical “do not eat me” signal between CD47 of tumor cells and signal-regulatory protein alpha (SIRPα) in macrophages^15^. In addition, recent research has demonstrated that targeting PD-1 and IL-2Rβγ with radiotherapy can lead to a durable local and systemic anti-tumor response in pancreatic cancer^16^. In addition, other studies have found that radiotherapy affects the metabolic regulation of the body. One study has demonstrated that the metabolism of lipids and amino acids not only meets the energy and biosynthesis needs of immune cells, but also regulates the survival, differentiation, and anti-tumor effects of immune cells by affecting cell signal transduction^17^. However, because these explorations have been limited to animal models and preclinical studies, more evidence and research efforts are needed to consolidate the findings. Personalized targeted neoantigen immunotherapy might potentially become the ultimate precision cancer treatment method^18^. Some novel targets, such as T-cell immunoglobulin and mucin domain 3 (Tim-3), T cell immunoreceptor with immunoglobulin and ITIM domain (TIGIT), and natural killer group 2 member D (NKG2D), also have broad application prospects. For instance, although many studies have shown little effect of blocking LAG-3 alone, the combination of relatlimab (anti-LAG-3) and nivolumab (anti-PD-1) exhibits promising clinical benefits, according to the RELATIVITY-047 study^19^. Thus, these alternative immune checkpoints may improve treatment efficacy and effectively overcome anti-PD-1 resistance.

**Figure 1:**
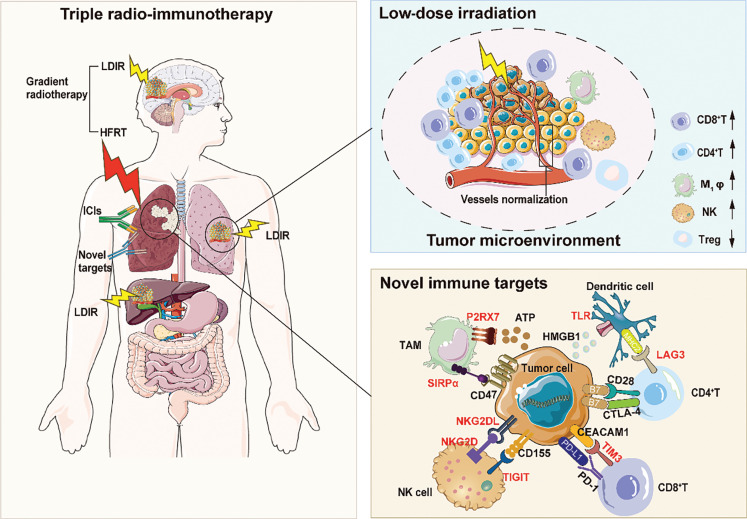
Mechanisms of triple radio-immunotherapy. The figure shows triple radio-immunotherapy including gradient radiotherapy, ICIs, and novel immune targets. Gradient radiotherapy comprises high-dose (HFRT) and low-dose radiotherapy (LDIR). LDIR reprograms the TME by increasing infiltration of CD4^+^ effector T cells, thus promoting M1 macrophage polarization, enhancing nature killer (NK) cell infiltration and cytotoxic function, and decreasing levels of immunosuppressive cytokines. Beyond the classical checkpoints, novel immune targets are distributed in a variety of cells, and include SIRPα and P2RX7 in macrophages; NKG2D and TIGIT in NK cells; TIM3 and LAG3 in T cells; and TLR in DC cells. These novel targets are highlighted in red. LDIR, low-dose irradiation; HFRT, hypofractionated radiotherapy; ICIs, immune checkpoint inhibitors; NK, natural killer; Treg, regulatory T cell; TAM, tumor-associated macrophage; DC, dendritic cell; ATP, adenosine 5′-triphosphate; MHCII, major histocompatibility complex II; HMGB1, high mobility group box-1 protein; TLR, Toll-like receptor; LAG3, lymphocyte activation gene-3; P2RX7, purinergic receptor P2X 7; SIRPα, signal-regulatory protein alpha; NKG2D, natural killer group 2 member D; NKG2DL, natural killer group 2 member D ligand; TIGIT, T-cell immunoreceptor with immunoglobulin and ITIM domain; PD-1, programmed cell death receptor-1; PD-L1, programmed cell death ligand-1; TIM3, T-cell immunoglobulin and mucin domain 3; CEACAM1, carcinoembryonic antigen related cell adhesion molecule 1; CTLA4, cytotoxic T lymphocyte-associated molecule-4; B7, B-lymphocyte activation antigen B7.

Finally, to overcome anti-PD-1 resistance, resistance mechanisms are another factor to consider. The anti-tumor effect of ICIs greatly depends on the TME content, and immune dysfunction is a major obstacle in ICI treatment. On the one hand, immune dysfunction represents the lack of immune cells in the TME. On the other hand, tumor infiltrated lymphocytes, particularly T cells, may became exhausted and unable to kill tumor cells effectively. Thus, breaking down the barrier of the TME and reversing T cell exhaustion might potentially overcome ICI resistance. The resistance mechanisms have been explored through analysis of the spatial characteristics of TME from patients resistant to anti-PD-1 therapy. The data have indicated that the effector lymphocytes cannot enter tumors, although immune cells accumulate and mobilize around tumors. A further investigation has indicated that CAFs play a crucial role in blocking immune cells from entering tumor tissue and exhibit a unique signature with high adherence especially high expression of FN1, COL11A1, COL1A1 and POSTN. This cluster of CAFs, defined as FAP^+^ α-SMA^+^ cells, has been described as a wall that obstructs infiltration of immune cells; these genes highly and specifically expressed in the CAFs have been termed “rampart genes”. Therefore, targeting rampart genes and CAFs may have favorable effects in overcoming immunotherapy resistance. Similarly, Lan and colleagues^20^ have demonstrated that simultaneous targeting of TGF-β/PD-L1 combined with radiotherapy attenuates the activity of CAFs, reprograms the TME, and increases tumor-infiltrating leukocytes. That study has also demonstrated the potential of triple radio-immunotherapy to overcome immunotherapy resistance. In conclusion, on the basis of the existing triple radio-immunotherapy including gradient radiotherapy and ICIs, the potential targets discussed above may be a promising triple therapy that may benefit more patients. The triple radio-immunotherapy is illustrated in **Figure 1**.

Several crucial issues regarding patient selection criteria, indications, and potential adverse effects for triple radio-immunotherapy must be noted. Clinical trials of triple radio-immunotherapy have enrolled populations of primarily treatment-naïve or treatment-refractory patients with metastatic solid tumors for the more malignant characteristics of cancer and a lack of effective treatments. An article on a standard framework for the qualifications of clinical trials of lung cancer, which is jointly completed by multiple FDA experts, emphasized disease stages and histological characteristics of stage IV (includes M1a, M1b and M1c stage diseases) in NSCLC, as the inclusion criteria. Oligometastases may have enhanced benefits from triple-immunotherapy. Regarding tolerance and safety, patients with oligometastatic disease can tolerate multi-site radiotherapy and may achieve curative outcomes through the synergetic systemic anti-tumor response potentiated by triple radio-immunotherapy. Another factor that should be considered is the expression of PD-L1. Several studies have demonstrated that PD-L1 expression in patients may be associated with the efficacy of ICIs. However, because the relationship between the expression level of PD-L1 and prognosis varies among tumors, further exploration remains needed. Although triple radio-immunotherapy may improve local and distant metastatic control, the clinical benefits may also come at a cost. Radiation-induced lung injury, cardiac injury, and esophageal injury influence the survival and therapeutic effects of patients with cancer. Moreover, ICIs may cause dermatologic toxicity, gastrointestinal toxicity, endocrinopathies, and pneumonitis through cytokine release syndrome. When patients receive triple radio-immunotherapy, toxicity concerns also increase. Mechanistically, radiotherapy induces release of inflammatory cytokines, which may aggravate ICI-associated adverse events and exert synergistic toxicity. However, no objective data suggest that simultaneous administration leads to a significant increase in toxicity. Thus, more clinical data from real world studies are needed to verify the safety of triple radio-immunotherapy.

## Perspectives

In the new era of iRT, the optimal combination treatment of ICIs and radiotherapy must urgently be identified. The goal of this editorial was to present the current evidence supporting triple therapy and to discuss promising novel targets to provide more therapeutic options. Furthermore, we believe that future research efforts to seek appropriate treatment approaches to cancer are just beginning to blossom and will benefit more patients in the future.
